# Authors' Response to the Commentary on “Micronized Vaginal Progesterone Dose and Serum Progesterone Thresholds Determine Reproductive Outcomes in Frozen–Thawed Embryo Transfer With Hormone Replacement Therapy”

**DOI:** 10.1002/rmb2.70089

**Published:** 2026-08-19

**Authors:** Ayumu Ito, Mami Sekiguchi, Yukiko Katagiri

**Affiliations:** ^1^ Department of Obstetrics and Gynecology, Faculty of Medicine Toho University Tokyo Japan; ^2^ Department of Obstetrics and Gynecology Toho University Omori Medical Center Tokyo Japan; ^3^ Reproduction Center Toho University Omori Medical Center Tokyo Japan

## Abstract

**Background:**

This response addresses the methodological concerns raised in a commentary on our study evaluating vaginal progesterone dose, serum progesterone concentrations, and reproductive outcomes in hormone replacement therapy–frozen embryo transfer cycles.

**Methods:**

We revisited the study objectives, analytical approach, interpretation of the proposed serum progesterone threshold, formulation‐specific considerations, treatment‐selection practices, and the potential influence of insurance reform.

**Main Findings:**

We clarify that the proposed serum progesterone threshold should not be interpreted as a definitive clinical cutoff and that differences among progesterone formulations and non‐random treatment selection may have influenced the observed associations. We also emphasize that several concerns raised in the commentary, including residual confounding and limitations inherent to the retrospective observational design, were acknowledged in the original manuscript.

**Conclusion:**

Our findings should be interpreted as exploratory observational associations rather than causal estimates. They may help generate hypotheses and inform the design of future prospective studies evaluating progesterone regimens and serum progesterone monitoring in hormone replacement therapy–frozen embryo transfer cycles.

We sincerely thank the commentators for their thoughtful appraisal of our study and for highlighting several important methodological considerations [1]. We appreciate the opportunity to further clarify the rationale, interpretation, and limitations of our work.

We are grateful for the commentators' insightful observations. However, we would like to emphasize that several of the concerns raised had already been recognized by us and explicitly discussed as limitations in the Discussion section of the manuscript [2]. Specifically, we acknowledged the retrospective nature of the study, the possibility of residual confounding, the limited discriminative ability of the identified serum progesterone threshold, and the fact that this threshold should not be interpreted as a definitive clinical target. Accordingly, we characterized our findings as exploratory and hypothesis‐generating rather than confirmatory.

We would also respectfully note that the primary objective of the study was clearly stated in both the Abstract and Introduction [2]. The aim was to evaluate the combined association of vaginal progesterone dose and achieved serum progesterone concentrations with reproductive outcomes in hormone replacement therapy–frozen embryo transfer (HRT‐FET) cycles. The study was not designed to estimate causal treatment effects within a formal causal inference framework. Rather, it was intended to evaluate clinically relevant associations in a large real‐world cohort.

Regarding the concern that the identified serum progesterone threshold may represent overfitting or may not be broadly generalizable, we largely agree. Our intention was not to establish a universally applicable clinical threshold, but rather to identify a statistically derived value associated with reproductive outcomes within our cohort. We therefore explicitly stated that external validation and prospective studies are necessary before any threshold can be adopted in routine clinical practice [2].

The commentators also raised concerns regarding pharmacokinetic differences among vaginal progesterone formulations and differences in blood sampling timing. These issues were specifically addressed in the Discussion [2]. Administration schedules, sampling times, and formulation‐specific pharmacokinetic characteristics were carefully considered when interpreting serum progesterone concentrations. Blood sampling schedules reflected routine institutional clinical practice rather than pharmacokinetic study protocols.

Nevertheless, the timing of blood sampling was generally consistent with the known pharmacokinetic profiles of the respective formulations. According to the package inserts, Lutinus and Luteum achieve stable plasma concentrations approximately 5 days after initiation of treatment [3, 4]. In contrast, OneCrinone reaches peak serum progesterone concentrations approximately 6 h after administration [5]. Therefore, although blood sampling was not prospectively standardized according to a pharmacokinetic protocol, the measured serum progesterone concentrations were obtained at clinically relevant time points and likely reflected meaningful pharmacological exposure to each formulation. These considerations were incorporated into the interpretation of our findings and discussed in the manuscript [2].

We also acknowledge the commentators' concern that the observed differences among the P(90), P(300), and P(800) groups may reflect formulation‐specific characteristics rather than progesterone dose alone. We agree that the evaluated regimens differed not only in nominal progesterone dose but also in pharmaceutical formulation, including vaginal gel, tablet, and capsule preparations. Importantly, this limitation was explicitly acknowledged in the Discussion [2]. We specifically noted that formulation characteristics and administration frequency may influence progesterone absorption and serum concentrations, making it difficult to isolate the effect of progesterone dose alone. Therefore, our findings should not be interpreted as demonstrating a pure dose effect independent of formulation. Rather, they reflect the combined clinical impact of the administered regimen, achieved serum progesterone exposure, and formulation‐specific characteristics in real‐world clinical practice.

Furthermore, regarding the choice of vaginal progesterone formulations, patients at our institution are informed of the report by Shiba et al. [6], which demonstrated no significant differences in assisted reproductive technology (ART) outcomes among currently available vaginal progesterone formulations. In addition, patients are provided with information regarding practical differences among formulations, including administration frequency, availability of applicators, and expected vaginal discharge characteristics. Patients are then allowed to select their preferred regimen. This clinical background should be considered when interpreting the present findings.

At the same time, it is important to note that previous studies have reported dose‐dependent improvements in ART outcomes within the same vaginal progesterone formulation [7, 8]. Therefore, although formulation‐specific characteristics should be considered when interpreting our findings, the possibility that progesterone dose itself contributes to differences in serum progesterone exposure and reproductive outcomes cannot be completely excluded. In addition, actual administration timing could not be independently verified because progesterone was self‐administered at home. We acknowledge that such variability may have influenced the measured serum progesterone concentrations. Because actual administration timing could not be independently verified, neither the magnitude nor the direction of any resulting measurement error can be determined. Furthermore, it is not possible to assess the extent to which deviations from the intended administration schedule may have affected serum progesterone measurements or the observed associations. This limitation is inherent to the retrospective real‐world nature of the present study.

With respect to the 600‐mg vaginal progesterone regimen, we would like to clarify that this group was not excluded after data collection. Rather, the comparative analyses focused on the 90‐mg, 300‐mg, and 800‐mg regimens because these were the three regimens with sufficient sample sizes in our clinical practice. As described above, patients selected their preferred regimen after receiving information regarding available formulations and their practical characteristics. Because the 600‐mg regimen requires more frequent administration and does not include an applicator, it was selected by relatively few patients. Consequently, the number of available cases was insufficient for robust statistical comparison. Therefore, analyses were focused on the three regimens with adequate sample sizes to ensure meaningful and interpretable comparisons.

The commentary further discusses exchangeability and the potential for treatment‐selection bias. We acknowledge that treatment allocation was not randomized. However, as described above, regimen selection at our institution was primarily based on patient preference after standardized counseling regarding the available progesterone formulations rather than on prognosis‐related clinical factors. Although residual selection bias cannot be completely excluded, this prescribing practice reduces the likelihood that treatment allocation was systematically determined by expected reproductive prognosis.

The commentators also questioned the selection of covariates and suggested a formal causal inference framework. We agree that causal inference methodologies, including DAG‐based approaches, provide valuable tools for estimating treatment effects. However, our study was not designed to estimate causal treatment effects. The selected covariates were chosen based on their established clinical relevance and previously reported associations with ART outcomes. Furthermore, the combined progesterone dose–serum progesterone classification remained an independent predictor of implantation, clinical pregnancy, ongoing pregnancy, and live birth after multivariable adjustment [2]. While residual confounding remains possible, these findings support the robustness of the observed associations within the limitations inherent to observational research.

While assumptions such as exchangeability, positivity, and consistency were not formally evaluated within a causal inference framework, we attempted to minimize bias through careful covariate adjustment based on established clinical knowledge and transparent reporting of study limitations. Accordingly, our findings should be interpreted as observational associations rather than estimates of causal treatment effects.

Finally, regarding the introduction of national insurance coverage for ART in Japan in April 2022 [9], we acknowledge that temporal changes in patient populations and clinical practice patterns may influence observational studies. However, the principal findings of the present study had already been observed in an internal interim analysis conducted before the implementation of national insurance coverage. Similar associations among progesterone dose, serum progesterone concentration, and reproductive outcomes, as well as a comparable serum progesterone threshold, were identified in pre‐insurance data and presented at the 75th Annual Congress of the Japan Society of Obstetrics and Gynecology in 2023. The consistency between the interim and final analyses suggests that the observed associations are unlikely to be explained solely by the insurance reform.

In conclusion, we appreciate the commentators' valuable insights and agree that our findings should be interpreted within the limitations inherent to retrospective observational research. At the same time, several of the issues raised, particularly those related to the retrospective design, formulation‐specific differences, and the exploratory nature of the proposed progesterone threshold, were already recognized and discussed in the manuscript [2]. We hope that our findings contribute to the ongoing discussion regarding individualized luteal phase support in HRT‐FET cycles and provide a foundation for future prospective investigations.

## Conflicts of Interest

The authors declare no conflicts of interest.

## Data Availability

The data that support the findings of this study are available on request from the corresponding author. The data are not publicly available due to privacy or ethical restrictions.
